# Learning from positive examples when the negative class is undetermined- microRNA gene identification

**DOI:** 10.1186/1748-7188-3-2

**Published:** 2008-01-28

**Authors:** Malik Yousef, Segun Jung, Louise C Showe, Michael K Showe

**Affiliations:** 1 Systems Biology Division, The Wistar Institute, Philadelphia, PA 19104, USA; 2 School of Biomedical Engineering, Science and Health Systems, Drexel University, Philadelphia, PA 19104, USA; 3 Computer Science, The College of Sakhnin, Sakhnin, Israel; 4 Sackler Institute of Graduate Biomedical Sciences, N.Y.U School of Medicine, New York, NY 10016, USA

## Abstract

**Background:**

The application of machine learning to classification problems that depend only on positive examples is gaining attention in the computational biology community. We and others have described the use of two-class machine learning to identify novel miRNAs. These methods require the generation of an artificial negative class. However, designation of the negative class can be problematic and if it is not properly done can affect the performance of the classifier dramatically and/or yield a biased estimate of performance. We present a study using one-class machine learning for microRNA (miRNA) discovery and compare one-class to two-class approaches using naïve Bayes and Support Vector Machines. These results are compared to published two-class miRNA prediction approaches. We also examine the ability of the one-class and two-class techniques to identify miRNAs in newly sequenced species.

**Results:**

Of all methods tested, we found that 2-class naive Bayes and Support Vector Machines gave the best accuracy using our selected features and optimally chosen negative examples. One class methods showed average accuracies of 70–80% versus 90% for the two 2-class methods on the same feature sets. However, some one-class methods outperform some recently published two-class approaches with different selected features. Using the EBV genome as and external validation of the method we found one-class machine learning to work as well as or better than a two-class approach in identifying true miRNAs as well as predicting new miRNAs.

**Conclusion:**

One and two class methods can both give useful classification accuracies when the negative class is well characterized. The advantage of one class methods is that it eliminates guessing at the optimal features for the negative class when they are not well defined. In these cases one-class methods can be superior to two-class methods when the features which are chosen as representative of that positive class are well defined.

**Availability:**

The OneClassmiRNA program is available at: [1]

## Background

MicroRNAs (miRNAs) are single-stranded, non-coding RNAs averaging 21 nucleotides in length. The mature miRNA is cleaved from a 70–110 nucleotide (*nt*) "hairpin" precursor with a double-stranded region containing one or more single-stranded loops. MiRNAs target messenger RNAs (mRNAs) for cleavage, primarily by repressing translation and causing mRNA degradation [2].

Several computational approaches have been applied to miRNA gene prediction using methods based on sequence conservation and/or structural similarity [3-7]. All of these methods rely on binary classifications that artificially generate a non-miRNA class based on the absence of features used to define the positive class. Nam, *et al*. [8] constructed a highly specific probabilistic Markov model (HMM) using the features of miRNA sequence and secondary structure; a negative class consisting of 1,000 extended stem-loop structures was generated based on several criteria, including sequence length (64–90 *nt*), stem length (above 22 *nt*), bulge size (under 15 *nt*), loop size (3–20 *nt*), and folding free energy (under -25 kcal/mol). Pfeffer, *et al*. [9] used support vector machines (SVMs) for predicting conserved miRNAs in herpes viruses. Features were extracted from the stem-loop and represented in a vector space. The negative class was generated from mRNAs, rRNAs, or tRNAs from human and viral genomes. The same technique was also applied to clustered miRNAs [10]. Xue, *et al*. [11] defined a negative class called pseudo pre-miRNAs. The criteria for this negative class included a minimum of 18 paired bases, a maximum of -15 kcal/mol folding free energy and no multiple loops. See [12] for a full review of miRNA discovery approaches.

In a recent publication we described a two-class machine learning approach for miRNA prediction using the naïve Bayes classifier [13]. Four criteria were used to select a pool of negative examples from candidate stem loops: stem length out of the range 42–85 *nt*, at most -25 kcal/mol of folding free energy, loop length greater than 26 *nt *and a number of base pairs (bp) that is not in the range (16–45) of the positive class. This approach, like all of the binary classifiers mentioned earlier, does not address the best number of negative examples to use and this influences the balance between false positive and false negative predictions. A comparison of a genuine negative class with one generated from random data for miRNA target prediction has been reported [14,15] showing that the two negative classes did not produce the same results.

Lately, Wang, *et al*. [16] developed an elegant algorithm, positive sample only learning (PSoL), to predict non-coding RNA (ncRNA) genes by generating an optimized negative class of ncRNA from so-called "unlabeled" data using two-class SVM. This method addresses predicting ncRNA genes without using negative training examples, but the procedure is quite complicated. Using their data set, we tested one of the one-class approaches, OC-SVM, to demonstrate a solution of the problem they addressed.

The method we now describe uses only the known miRNAs (positive class) to train the miRNA classifier. We emphasize that the one-class approach is a good tool not only for its simplicity, but in order to avoid generating a negative class where the basis for defining this class is not clear. The only required input for this tool is the miRNA sequences from a specific genome (or multiple genomes) for building the model to be used later as a miRNA predictor. In addition, we have tested the accuracy of the one-class method in the identification of miRNA in "newly sequenced" organisms such as the *Epstein Barr virus *genome, which were not used for training the classifier. The results are comparable to our two-class approach with high sensitivity and similar numbers of new predictions.

## Results

### Performance evaluation

Table 1 shows the performance of five one-class classifiers as well as two-class naïve Bayes and two-class SVM for comparison. The results of the one-class approaches show a slight superiority for OC-Gaussian and OC-KNN over the other one class methods based on the average of the MCC measurement. However, accuracy is less than the two-class approaches by about ~8%–10%. During the training stage of the one-class classifier we have set the 10% of the positive data, whose likelihood is furthest from the true positive data based on the distribution, as "outliers" in order to produce a compact classifier. This factor might cause a loss of 10% of information about the target class which might also result in reducing performance compared to the two class approach.

**Table 1:** One-class results obtained from the secondary features plus sequence features.

	*C. elegans*			*Mouse*			*Human*			*All-miRNA*			
	
Method	Sen	Spe	MCC	Sen	Spe	MCC	Sen	Spe	MCC	Sen	Spe	MCC	Average MCC
OC-SVM	0.73	0.93	0.67	0.80	0.93	0.74	0.72	0.99	0.74	0.69	0.91	0.62	0.70
OC-Gaussian	0.84	0.93	0.77	0.89	0.93	0.82	0.82	0.99	0.82	0.82	0.99	0.82	0.81
OC-Kmeans	0.79	0.93	0.73	0.85	0.92	0.77	0.89	0.92	0.81	0.89	0.80	0.69	0.75
OC-PCA	0.87	0.89	0.76	0.88	0.92	0.80	0.90	0.79	0.69	0.90	0.86	0.76	0.77
OC-KNN	0.90	0.86	0.76	0.90	0.92	0.82	0.90	0.96	0.86	0.90	0.93	0.83	0.82

Two-Class													

Naïve Bayes	0.89	0.93	0.82 (125)	0.93	0.97	0.90 (200)	0.99	0.92	0.92 (300)	0.97	0.96	0.93 (4000)	0.88
SVM	0.90	0.97	0.87 (200)	0.95	0.98	0.93 (500)	0.99	0.99	0.98 (300)	0.98	0.95	0.93 (900)	0.92

Sen = sensitivity, Spe = specificity, and MCC = Matthews Correlation Coefficient. Results are presented for four genomes individually (*C. elegans, Mouse, and Human*) and *All-miRNA *as a mixture of multiple miRNAs species. The number in parentheses is the corresponding number of optimal negative examples giving the highest MCC.

Xue, *et al *[11] reported a sensitivity of 0.93 and specificity of 0.88 using two-class SVM on the human miRNA with the same number of negative examples (1,000) as we used. Computing the MCC for their results gives MCC = 0.81. OC-KNN with the same data *(Human) *achieves slightly better results with MCC = 0.86 while comparable results are obtained with OC-Gaussian. See the column "MCC" under *Human *and the rows of "OC-Gaussian" and "OC- KNN" in Table 1. The two-class implementations in Table 1 are also superior with *Human *(MCC = 0.98 for SVM and MCC = 0.92 for naïve Bayes).

Nam, *et al *[8] used a hidden Markov model (HMM) to classify the human miRNA along with 1,000 negative examples to estimate the performance of their approach. They report 0.73 for sensitivity and 0.96 for specificity (MCC = 0.71). All the OC-methods outperform this algorithm except the OC-SVM which is about the same.

### Comparison with other prediction methods

The aim of this section is to evaluate the performance of the one-class classification considering different features suggested by other studies [10,11,17]. We used the MCC measurement for comparison purposes.

The triplet-SVM classifier is a 2-class tool developed by Xue, *et al *[11] that does not rely on comparative genomic approaches. The data consist of training and testing set and these were used to evaluate the performance of one-class approaches. We used the positive 163 human pre-miRNAs for training and then tested with the 30 human pre-miRNAs as positive and 1,000 pseudo pre-miRNAs as negative class. The different performances of one-class approaches are presented in Table 2. Many of the results have higher sensitivity but lower specificity than the 2-class, although some of the difference may be attributable to the different feature set. However, two-class naïve Bayes and two-class SVM (using our features) outperform these results by about 11% and 17% respectively based on the MCC measurement with *Human *miRNAs in Table 1.

**Table 2:** One-class results obtained from triplet-SVM and RNAmicro1.1 tools based on their specific features.

	triplet-SVM (*Human*)			RNAmicro1.1		
	
Method	Sen	Spe	MCC	Sen	Spe	MCC
OC-SVM	0.93	0.78	0.72	0.93	0.94	0.87
OC-Gaussian	0.90	0.88	0.78	0.90	0.96	0.87
OC-Kmeans	0.98	0.8	0.79	0.93	0.92	0.84
OC-PCA	0.97	0.79	0.77	0.90	0.96	0.86
OC-KNN	0.93	0.84	0.77	0.91	0.95	0.87
Original study results	0.93	0.88	0.81	0.84	0.99	0.84

The last row has the originally reported results.

RNAmicro1.1 is another miRNA prediction tool developed by Hertel and Stadler [17] that relies mainly on comparative sequence analysis using two-class SVM. The positive set includes 295 alignments of distinct miRNA families obtained from the union of animal miRNAs contained in the Rfam 6.0 (276 are considered with the refined list provided by the authors). The negative set (about 10,000 provided as a new list by the authors) is constructed mainly from tRNA alignments. We have chosen randomly 1,000 to match the same size of negative class used by us and other studies. The results of one-class approaches (Table 2) are comparable (an advantage for most of the one-class methods of about 3% from the results reported by the authors). As observed earlier, two-class naïve Bayes and two-class SVM (based on our features) outperform these results by about 9% with similar data (*All-miRNA*).

PSoL is an iterative method developed by Wang, *et al*. [16] to predict ncRNA genes from the *E. coli *genome and to define an optimized negative class using two-class SVM. It selects an initial negative set from an unlabelled set, and then uses two-class SVM to expand the negative set gradually by reassigning from the unlabelled data. The expansion is continued until the remaining unlabeled set is reduced to a predefined size *N *and this set is considered to be positive predictions. We used the same data as the authors used in their study – 321 positive examples along with 11,818 unlabeled examples – for the comparison with OC-SVM using linear kernel. We followed their assessment steps using 5-fold cross validation. OC-SVM reached a sensitivity of 0.73 with specificity of 0.92. This is comparable to the PSoL recovery rate (sensitivity) of about 0.8 when the expansion is stopped at *N *= 1,000.

### Predicting miRNA genes in the *Epstein Barr Virus (EBV) *genome

**Figure 1:**
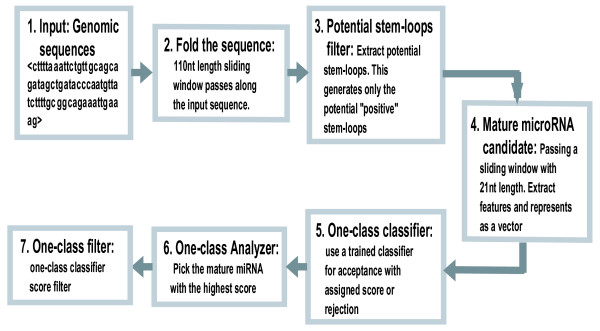
Components of the one-class computational procedure.

*The EBV *genome has been extensively studied [9,18,19] and an estimate of 20–30 *EBV *miRNAs has been reported. However, additional miRNA may remain to be discovered in the *EBV *genome. We downloaded the whole genome of the *Epstein Barr virus *(Human herpes virus 4, NC_007605 version NC_007605.1 GI: 82503188) with length of 171,823 *nt*, from the NCBI website [20], and passed it through the pipeline shown in Fig. 1, which is similar to the one used in Yousef et al. [13]. Thirty-two mature miRNAs reported in Rfam [21] (Release 8.1: May 2006) were used to estimate the sensitivity of each trained type of classifier (Table 3). As a comparison with the two-class approach, the same experiment was carried out using the BayesMiRNAfind classifier [13]. We generated 5,207 candidates at step 2 (Fig. 1) but only 1,251 passed the potential stem-loop filter at step 3. At step four 68,702 mature miRNA candidates were produced from the 1,251 pre-miRNA candidates.

**Table 3:** Prediction of miRNAs in *Epstein Barr Virus *with the one-class methods.

Train	*All-miRNA*		*Mouse*		*Human*		Recent *Human*	
	
	Sen	New	Sen	New	Sen	New	Sen	New
OC-SVM	0.84 (27/32)	236	0.72 (23/32)	236	0.81 (26/32)	279	0.94 (30/32)	198
OC-Gaussian	0.88 (28/32)	258	0.81 (26/32)	233	0.81 (26/32)	266	0.84 (27/32)	275
OC-Kmeans	0.90 (29/32)	284	0.97 (31/32)	266	0.78 (25/32)	269	0.97 (31/32)	271
OC-PCA	0.97 (31/32)	284	0.90 (29/32)	255	0.90 (29/32)	259	0.94 (30/32)	283
OC-KNN	0.88 (28/32)	272	0.84 (27/32)	266	0.81 (26/32)	283	0.91 (29/32)	269

naïve Bayes	0.84 (27/32)	165	N/A	N/A	N/A	N/A	0.94 (30/32)	276

*All-miRNA*, *Mouse*, or *Human *served as training data sets. New = new miRNA predictions.

As shown in Table 3, all the one-class methods are able to recognize most of the reported virus miRNA with sensitivity of 72%–90%. OC-PCA has the highest sensitivity when trained by *All-miRNA *or *Human *miRNAs, whereas OC-Kmeans is superior when trained by *Mouse *miRNAs. BayesMiRNAfind succeeds in achieving 84% sensitivity along with 165 reported new predictions.

Rfam miRNAs registry Release 8.1: (May 2006) [21] includes a new list of human miRNA (462 stem-loop sequences) and we also used this new data to train the one-class methods. These results are presented in the last column of Table 3. In this study, 18 of the 462 new human miRNAs were discarded since they fail to form a stem-loop structure based on mfold. The new one-class results with this data set are better than those determined with the previous list of human miRNAs or to the other data sets included in Table 3. We believe this is because the "recent human " list is richer and cleaner as the number of miRNAs listed is almost double the previous one, and it is not surprising that the performance of classifiers improves as the number of positive examples for training increases. The two-class BayesMiRNAfind was also retrained with the new human miRNA sequences and with different numbers of negative examples. The best results obtained were with 200 negative examples yielding 94% (30/32) sensitivity along with 276 new miRNA predictions.

**Figure 2:**
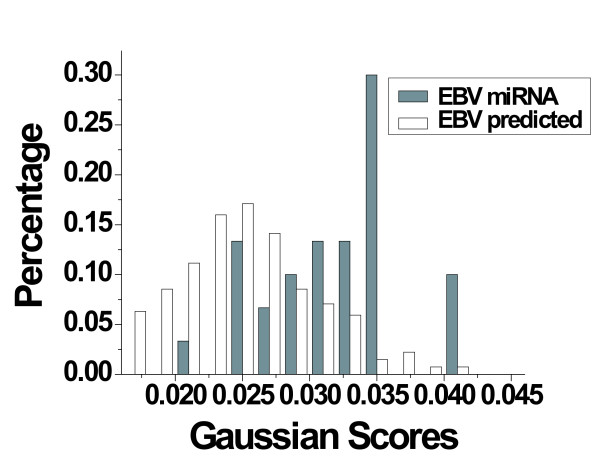
**One-Class Gaussian classification scores**. This shows the distribution of OC-Gaussian classifier scores over the miRNAs class and the new miRNA prediction from EBV genome sequences. *All-miRNA *is used for training.

Generally, approximately 4% of the new miRNA candidates (~200/5,207) were identified by the computational procedure (Fig. 1, compare step 6 with step 3) while about 88% (28/32) of the known miRNAs were retrieved (Table 3). Using different filters (score, conservation, common, etc,) can reduce the number of miRNA predictions; for example, selecting 0.25 as a threshold (step 7 in Fig. 1) for OC-Gaussian with *All-miRNA *model (See Fig. 2) will recover 97% of the captured true miRNA (0.97*28) while reducing the new miRNA prediction by 42%. A threshold of 0.3 recovers 40% of the captured miRNA (0.4*28) and a reduction of about 95% of the new miRNA predictions. The choice of the threshold is arbitrary and it determines the number of the final predictions. However, one can set a threshold that captures 70–80% of the true miRNA to have reliable predictions. To assess our predictions we have used the triplet-SVM classifier tools [11] to evaluate the OC-Gaussian results. 87% of the known miRNA captured by OC-Gaussian classifier were confirmed by the triplet-SVM classifier and 13% of our new miRNA predictions were confirmed as well. This interesting result suggests that combining different methods may lead to classifying miRNAs more accurately. This also may strengthen our main purpose: to reduce the false positive predictions while obtaining high sensitivity when analyzing a large genomic sequence.

## Conclusion

The one-class approach in machine learning has been receiving more attention particularly for solving problems where the negative class is not well defined [22-25]; moreover, the one class approach has been successfully applied in various fields including text mining [26], functional Magnetic Resonance Imaging (fMRI) [27] and signature verification [28].

In this paper we have presented a one-class approach to predicting miRNAs based on their secondary structure and sequence features from other studies using information only from the positive (miRNA) class. We approached this problem because an arbitrary selection of the negative class in these predictive studies can be difficult and can bias the results. This may be particularly true as new organisms are surveyed where the examples for a negative class are not clearly defined. We find that the accuracy of prediction using one-class methods depends on the features used and in some cases may be better than a two-class approach judged by our own and others' studies. We found slightly greater accuracy for 2-class than one-class using our feature set, but this was not generally true using different feature sets (see Table 2).

We find that the miRNA features used in our studies appear to describe the miRNA class more accurately than those used in some previous studies [11,17]. The features we proposed are more likely to capture the functionality of the miRNA by considering the bulges, loops and asymmetric-loops features. We also show that the triplet-SVM classifier tools [11] combining with some classifiers (either one-class or two-class) using our suggested features is a reasonable way to reduce the false positive prediction while preserving high sensitivity. This approach could be usefully applied to a large genome (as human, mouse, and etc.), especially when conservation is not considered as a feature for a cross-species analysis.

Among the different one-class approaches including Support Vector Machines (SVMs), Gaussian, Kmeans, Principal Component Analysis (PCA), and K-Nearest Neighbor (K-NN), we found that OC-KNN and OC-Gaussian are superior to others in terms of prediction specificity as measured by their ability to accurately capture only the known miRNAs. High specificity is very important in genome wide analyses where the numbers of predictions can be very large and false positives must be minimized.

The principal advantage to the one class approach lies in not having to define the characteristics of a negative class. Two-class classifiers are an obvious choice in many instances where the negative class is obvious, e.g., comparison of tissue from healthy controls with tumor tissue from a cancer patient. When searching a genome for miRNA, the definition of non-miRNA is not well defined so many false positives may be predicted and some true miRNA species may not be detected. We have applied this one-class approach to miRNA discovery, and a similar application might also be useful for miRNA target prediction in which the definition of a negative class is also ambiguous.

## Methods

### Choosing structural and sequence features

We begin by describing features of miRNA extracted from both secondary structure and sequences. We adopted the structural features from our two-class miRNA prediction method [12] for the development of a one-class method. For the positive (miRNA) class, the 21 nt of the mature miRNA are mapped into its associated stem-loop (generated by the mfold program [29]) and then features are extracted as described below. Similarly, we used sliding 21 nt windows along each stem-loop strand to extract features for the negative (non-miRNA) class.

**Figure 3:**
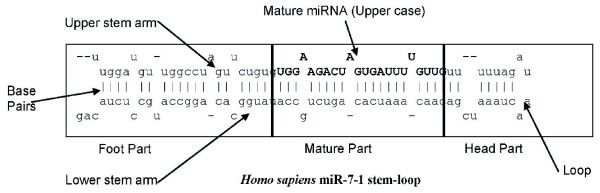
**Partition stem-loop into 3 parts**. Foot, Mature and Head features to determine potential stem-loops.

For the structural features, 62 features are derived from three parts of the associated hairpin (stem-loop) (See Fig. 3) – foot, mature, and head – and include the following for each of these parts: (1) the total number of base pairs(bp), (2) the number of bulges, (3) the number of loops, (4) the number of asymmetric loops, (5) eight features representing the number of bulges of lengths 1–7 and greater than 7, (6) eight features representing the number of symmetric loops of length 1–7 and greater than 7, (7) the distance from the mature miRNA candidate to the first paired base of the foot and head part.

For the sequence features, we define "words" as sequences having lengths equal to or less than 3. The frequency of each word in the first 9 nt of the 21 nt putative mature miRNA is extracted to form a representation in the vector space. For justification of the use of first 9 nt and the 1- 2- and 3-mers ("words"), a comparison study between different "words" lengths was conducted as presented in Table A and Table B [Additional file 1]. More detailed information can be found in [13]. When using a two-class method, we chose values for features of the negative class which lie outside the distributions of values for those features which characterized the positive class [13]. For one-class methods this required arbitrary choice is unnecessary since there is no need to describe a negative class.

### One-class methods

In general a binary learning (two-class) approach to miRNA discovery considers both positive (miRNA) and negative (non-miRNA) classes by providing examples for the two classes to a learning algorithm in order to build a classifier that will attempt to discriminate between them. The most common term for this kind of learning is *supervised learning *where the labels of the two-classes are known before hand. One-class uses only the information for the target class (positive class) building a classifier which is able to recognize the examples belonging to its target and rejecting others as outliers.

Among the many classification algorithms available, we chose five one-class algorithms to compare for miRNA discovery. We give a brief description of each one-class classifier and we refer references [30,31] for additional details including a description of parameters and thresholds. The LIBSVM library [32] was used as implementation of the SVM (both one-class and two-class using the RBF kernel function) and the DDtools [33] for the other one-class methods. See Table D [Additional file 1] for optimal parameter selections and used parameter value.

Each classifier returns a score which is a measure of the likelihood that the candidate being tested belongs to the positive class. The highest score determines the preferred candidate associated with a given hairpin structure, see Fig. 1

### One-class support vector machines (OC-SVM)

Support Vector Machines (SVMs) are a learning machine developed as a two-class approach [34,35]. The use of one-class SVM was originally suggested by Scholkopf et al. [31]. One-class SVM is an algorithmic method that produces a prediction function trained to "capture" most of the training data. For that purpose a kernel function is used to map the data into a feature space where the SVM is employed to find the hyper-plane with maximum margin from the origin of the feature space. In this use, the margin to be maximized between the two classes (in two-class SVM) becomes the distance between the origin and the support vectors which define the boundaries of the surrounding circle, (or hyper-sphere in high-dimensional space) which encloses the single class.

### One class Gaussian (OC-Gaussian)

The Gaussian model is considered as a density estimation model. The assumption is that the target samples form a multivariate normal distribution, therefore for a given test sample *z *in *n*-dimensional space, the probability density function can be calculated as:

$$p(z)=\frac{1}{{\left(2\Pi \right)}^{n/2}{\left|\Sigma \right|}^{1/2}}e^{\left(-1/2{\left(z-\mu \right)}^{T}\Sigma^{-1}(z-\mu \right)}$$

where *μ *and *Σ *are the mean and covariance matrix of the target class estimated from the training samples.

### One-class Kmeans (OC-Kmeans)

Kmeans is a simple and well-known unsupervised machine learning algorithm used in order to partition the data into *k *clusters. Using the OC-Kmeans we describe the data as *k *clusters, or more specifically as *k *centroids, one derived from each cluster. For a new sample, *z*, the distance *d*(*z*) is calculated as the minimum distance to each centroid. Then based on a user threshold, the classification decision is made. If *d*(*z*) is less than the threshold the new sample belongs to the target class, otherwise it is rejected.

One-class principal component analysis (OC-PCA). Principal component analysis (PCA) is a classical statistical method known as a linear transform that has been widely used in data analysis and compression. Mainly PCA is a projection method used for reducing dimensionality in a given dataset by capturing the most variance by a few orthogonal subspaces called principal components (PCs). For the one-class approach (OC-PCA) one needs to build the PCA model based on the training set and then for a given test example *z *the distance to the PCA(*z*) model is calculated and used as a decision factor for acceptance or rejection.

### One-class K-nearest neighbor (OC-KNN)

The one-class nearest neighbor classifier (OC-KNN) is a modification of the known two-class nearest neighbor classifier which learns from positive examples only. The algorithm operates by storing all the training examples as its model, then for a given test example *z*, the distance to its nearest neighbor *y *(*y *= *NN*(*z*)) is calculated as *d*(*z*, *y*). The new sample belongs to the target class when:

$$\frac{d(z,y)}{d(y,NN(y))}<\delta$$

where *NN*(*y*) is the nearest neighbor of *y*, in other words, it is the nearest neighbor of the nearest neighbor of *z*. The default value of *δ *is 1. The average distance of the *k *nearest neighbors is considered for the OC-KNN implementation.

### Classification performance evaluation

To evaluate classification performance, we used the data generated from the positive class and 1,000 negative examples chosen at random from the negative class pool (candidates which failed one of four initial criteria, as previously described [13]). The negative class is not used for training of the one-class classifiers, but merely for estimating the specificity performance.

The positive class data includes 117 miRNAs from *C. elegans*, 224 miRNAs from *Mouse*, 243 miRNAs from *Human*, and all 1,359 known miRNAs from other species, called *All-miRNA *[13]. In *All-miRNA*, 100 homologous precursors were removed from the dataset to avoid bias, but this had little effect on accuracy (compare Table F with Table G [Additional file 1]). See [13] for more details.

The two-class naïve Bayes classifier and two-class SVM were trained with 90% of the positive miRNA data and with a negative class ranging from 50 examples to 900 chosen randomly from the pool of 1,000 negative examples. The test was done with the remaining 10% from the miRNA class and the remaining negative examples. The evaluation procedure was repeated 100 times and the results are reported in Table 1 under the title "Two-Class." For the naïve Bayes test with the set *All-miRNA*, the number of negative examples was extended to 55,000.

Each one-class algorithm was trained using 90% of the positive class and the remaining 10% was used for sensitivity evaluation. The randomly selected 1,000 negative examples were used for the evaluation of specificity. The whole process was repeated 100 times in order to evaluate the stability of the methods. Additionally, the Matthews Correlation Coefficient (MCC) [36] measurement is used to take into account both over-prediction and under-prediction in imbalanced data sets. It is defined as:

$$MCC=\frac{\left(TpTn-FpFn\right)}{\sqrt{\left(Tp+Fp)(Tp+Fn)(Tn+Fn)(Tn+Fp\right)}}$$

The MCC score is in the interval (-1, 1), where one shows a perfect separation, and zero is the expected value for random scores.

In Table 1, we present the performance for each one-class classifier (The performance using secondary structural features without any sequence information is shown separately in Table H [Additional file 1]). The performance for the two-class methods is presented as well. The results for a specific number of negative examples with the highest MCC only are shown.

## Authors' contributions

MY originated the project, supervised programming and drafted the paper, SJ carried out calculations and programming, MKS and LCS provided the biological applications, reviewed data and finalized manuscript. All authors read and approved the final manuscript.

## Supplementary Material

Additional File 1 Annotation of species used and additional data on accuracy associated with various one-class parameters. **Table A. **Sensitivity (Sen) and specificity (Spe) from one-class SVM using various word-lengths and the first 9 nt of the mature miRNA. **Table B. **Sensitivity (Sen) and specificity (Spe) obtained from one-class SVM to find the optimal number of the first *k *nucleotides using word length 3/4 3. **Table C. **Importance of the sequence features alone for classification. **Table D. **Optimized parameters for each one-class method. **Table E. **Annotation for all used species. **Table F. **The size of each dataset after removing similar structures of mature microRNAs. **Table G. **Accuracy in classification of *All-miRNA *dataset after masking to remove homologs. **Table H. **One-Class results obtained from the secondary features only and secondary features plus sequence features Click here for file
