# Supplementary material for: Learning from positive examples when the negative class is undetermined- microRNA gene identification
Source: Algorithms Mol Biol. 2008 Jan 28;3:2. doi: 10.1186/1748-7188-3-2 (PMC2248178; doi:10.1186/1748-7188-3-2)
Supplement: Additional File 1 — Annotation of species used and additional data on accuracy associated with various one-class parameters. Table A. Sensitivity (Sen) and specificity (Spe) from one-class SVM using various word-lengths and the first 9 nt of the mature miRNA. Table B. Sensitivity (Sen) and specificity (Spe) obtained from one-class SVM to find the optimal number of the first k nucleotides using word length 3/4 3. Table C. Importance of the sequence features alone for classification. Table D. Optimized parameters for each one-class method. Table E. Annotation for all used species. Table F. The size of each dataset after removing similar structures of mature microRNAs. Table G. Accuracy in classification of All-miRNA dataset after masking to remove homologs. Table H. One-Class results obtained from the secondary features only and secondary features plus sequence features [file 1748-7188-3-2-S1.doc]

**Additional file:**

The following tables — Table A, Table B, and Table C — compare the relative importance of nucleotides in the mature miRNA sequence as features for classification. In each case, we counted the frequency of all of the words (mers) of length 3, 2, or 1 base. Among the five methods used in our study, we selected one-class SVM (OC-SVM) for this experiment. The OC-SVM algorithm was trained with 90% of positive class data from Rfam — *C.elegans*, *Mouse,* or *All-miRNA* which is a mixture of all the species shown in Table D — respectively and tested by combining the remaining 10% of the positive class data with the 10,000 negative examples randomly selected from the negative class pool. The negative class pool was generated using 300 random sequences highly conserved between mouse, rat, human, dog, and chicken (using multiple alignments May 2004 downloaded from UCSC [4].  By applying the third and the fourth stages of the computational procedure [5] on those sequences, 239,674 stem-loops were generated to serve as a pool of negative examples. These stem-loops fail at least one of the four miRNA structural criteria derived from stem length, loop length, paired bases, and folding free energy.

**Table A.** Sensitivity (Sen) and specificity (Spe) from one-class SVM using various word-lengths and the first 9 nt of the mature miRNA. The purpose of Table A is to find the optimal word-length to extract all the important sequence features using the first 9 nt of the mature miRNA. *All k-mer* includes all the word lengths from 1 to *k* and *only k-mer* stands for only word length *k*. True negative percentages are highest with *k*=1 (simple base composition). True positive for the individual species peak at *k*=3, but for *All-miRNA*, *k*=1 is also most efficient. No clear advantage between all *k-mer* and only *k-mer* were found based on the results at *k*=3. However, we decided to use the *all k-mer* since it has slightly better specificity which is important when dealing with a whole genome. Similar results were found with other classifiers.

| *k* | Secondary & sequence | | | | | | | | | | | |
| --- | --- | --- | --- | --- | --- | --- | --- | --- | --- | --- | --- | --- |
|  | *C.elegans* | | | | *Mouse* | | | | *All-miRNA* | | | |
|  | *all k-mer* | | *only k-mer* | | *all k-mer* | | *only k-mer* | | *all k-mer* | | *only k-mer* | |
|  | Sen | Spe | Sen | Spe | Sen | Spe | Sen | Spe | Sen | Spe | Sen | Spe |
| 1 | 0.54 | 0.98 | 0.54 | 0.98 | 0.70 | 0.97 | 0.70 | 0.97 | 0.76 | 0.92 | 0.76 | 0.92 |
| 2 | 0.56 | 0.97 | 0.58 | 0.97 | 0.72 | 0.97 | 0.73 | 0.96 | 0.74 | 0.92 | 0.75 | 0.91 |
| 3 | 0.70 | 0.93 | 0.69 | 0.93 | 0.79 | 0.93 | 0.80 | 0.92 | 0.62 | 0.94 | 0.64 | 0.92 |
| 4 | 0.61 | 0.92 | 0.62 | 0.92 | 0.59 | 0.94 | 0.63 | 0.92 | 0.40 | 0.93 | 0.44 | 0.91 |
| 5 | 0.44 | 0.93 | 0.46 | 0.92 | 0.51 | 0.94 | 0.51 | 0.93 | 0.30 | 0.93 | 0.29 | 0.94 |

**Table B.** Sensitivity (Sen) and specificity (Spe) obtained from one-class SVM to find the optimal

number of the first *k* nucleotides using word length ≤ 3. The purpose of Table B is to decide how many bases of the mature miRNA to use, with *all 3-mer* as the representation of the sequence features. The table represents the sensitivity (Sen) and the specificity (Spe) with different genomes based on distinctive number of nucleotide and shows that the first 9-nucleotides of the mature miRNA are valid to extract the important sequence features. The specificity increases while the sensitivity decreases as *k-nucleotide* increases for the individual genomes. In contrast to the two genomes the sensitivity of *All-miRNA* increases along with *k*, whereas the specificity has no significant changes. *All-miRNA* shows a slight continuing increase in accuracy when using the entire mature sequence but we decided to use the first 9 nt out of 21 nt in our study since it is known for having most of the biological information. Better accuracy would be obtained using *All-miRNA* for training if the entire mature sequence were used, at a cost of increased time for calculation.

| *k*-nucleotide | Secondary & sequence | | | | | |
| --- | --- | --- | --- | --- | --- | --- |
|  | *C.elegans* | | *Mouse* | | *All-miRNA* | |
|  | Sen | Spe | Sen | Spe | Sen | Spe |
| 3 | 0.74 | 0.90 | 0.83 | 0.90 | 0.66 | 0.90 |
| 4 | 0.75 | 0.90 | 0.83 | 0.91 | 0.66 | 0.91 |
| 5 | 0.74 | 0.91 | 0.83 | 0.92 | 0.69 | 0.91 |
| 6 | 0.75 | 0.92 | 0.82 | 0.93 | 0.66 | 0.90 |
| 7 | 0.72 | 0.92 | 0.81 | 0.93 | 0.65 | 0.91 |
| 8 | 0.73 | 0.92 | 0.80 | 0.94 | 0.69 | 0.91 |
| 9 | 0.73 | 0.92 | 0.79 | 0.93 | 0.69 | 0.91 |
| 11 | 0.70 | 0.93 | 0.77 | 0.93 | 0.69 | 0.90 |
| 13 | 0.69 | 0.93 | 0.77 | 0.94 | 0.68 | 0.91 |
| 15 | 0.67 | 0.94 | 0.75 | 0.93 | 0.68 | 0.91 |
| 17 | 0.65 | 0.94 | 0.77 | 0.93 | 0.68 | 0.91 |
| 19 | 0.63 | 0.95 | 0.76 | 0.95 | 0.71 | 0.92 |
| 21 | 0.60 | 0.95 | 0.77 | 0.95 | 0.71 | 0.91 |

**Table C.**  Importance of the sequence features alone for classification. Sensitivity (Sen) and specificity (Spe) are obtained from the one-class SVM to examine performance based on the sequence features only. The parameter *nu* is a width factor of the RBF kernel in SVM examining greedy values, decreasing a density. We use the first 9 nucleotides (of the

mature miRNA) with word length ≤ 3. The purpose of Table C is to examine the importance of the sequence features when the secondary features are excluded. Table C shows that sequence features alone are not able to achieve good results. The best specificity of performance is too low to be useful.

| *nu* | Sequence | | | | | |
| --- | --- | --- | --- | --- | --- | --- |
|  | *C.elegans* | | *Mouse* | | *All-miRNA* | |
|  | Sen | Spe | Sen | Spe | Sen | Spe |
| 0.5 | 0.45 | 0.70 | 0.47 | 0.70 | 0.49 | 0.67 |
| 0.4 | 0.55 | 0.65 | 0.59 | 0.61 | 0.56 | 0.61 |
| 0.3 | 0.63 | 0.57 | 0.68 | 0.53 | 0.71 | 0.52 |
| 0.2 | 0.75 | 0.48 | 0.77 | 0.40 | 0.77 | 0.43 |
| 0.1 | 0.83 | 0.37 | 0.87 | 0.27 | 0.85 | 0.28 |
|  |  |  |  |  |  |  |
| 0.05 | 0.88 | 0.33 | 0.91 | 0.24 | 0.91 | 0.21 |
|  |  |  |  |  |  |  |

**Table D.** Optimized parameters for each one-class method. *N* for OC-SVM is a width factor of the RBF kernel, *r* for Gaussian is to regulate an estimated covariance matrix; *k* for OC-Kmeans is the initial group centroid; *n* for OC-PCA stands for a number of features. With the three given data sets, a common fraction for target and outlier, which are 0.9 and 0.1 respectively, is used for all the classifiers — OC-SVM, OC-Gaussian, OC-Kmeans, OC-PCA, and OC-KNN. The same optimal parameter, was found for OC-Gaussian, OC-Kmeans, and OC-PCA method for the two species — *C.elegans* and *Mouse,* but different for *All-miRNA*.

| Method | *C.elegans* | | *Mouse* | | *All-miRNA* | |
| --- | --- | --- | --- | --- | --- | --- |
|  | Secondary | Secondary&  Sequence | Secondary | Secondary& Sequence | Secondary | Secondary& Sequence |
| SVM (*n*) | 0.05 | 0.005 | 0.05 | 0.005 | 0.2 | 0.3 |
| Gaussian (*r*) | 0.1 | 0.8 | 0.1 | 0.8 | 0.01 | 0.1 |
| Kmeans (*k*) | 15 | 7 | 15 | 7 | 15 | 12 |
| PCA (*n*) | 0.8 | 0.7 | 0.8 | 0.7 | 0.8 | 0.7 |
| KNN (*k*) | 1 | 1 | 1 | 1 | 1 | 1 |

**Table E.** Annotation for all used species

| Family | Sub-family | | | initial | species name |
| --- | --- | --- | --- | --- | --- |
| Metazoa  (animals) | Arthropoda | | | dme | Drosophila melanogaster |
| dps | Drosophila pseudoobscura |
| Nematoda | | | cel | [Caenorhabditis elegans](http://microrna.sanger.ac.uk/sequences/help/summary.shtml#cel%23cel) |
| cbr | Caenorhabditis briggsae |
| Vertebrata | Aves (bird) | | gga | Gallus gallus |
| Mammalia | [Primates](http://microrna.sanger.ac.uk/sequences/help/summary.shtml#primates%23primates) | hsa | Homo sapiens |
| Rodentia | mmu | [Mus musculus](http://microrna.sanger.ac.uk/sequences/help/summary.shtml#mmu%23mmu) |
| rno | Rattus norvegicus |
| Pisces (fish) | | | dre | Danio rerio |
| Viridiplantae  (plants) |  | | | zma | Zea mays |
| osa | Oryza sativa |
| ath | Arabidopsis thaliana |

**Table F.** The size of each dataset after removing similar structures of mature microRNAs. *All-miRNA* dataset is the one with all species combined. Many similar structures based on homology are found so that we removed them to avoid bias on the test of sensitivity and specificity. Many of the known miRNAs are homologs and very similar in their seed part. We use the Euclidean distance (ED) of each feature on vector space to see the similarity between known miRNAs. If the ED is less or equal than 0.05, we exclude the miRNA from its dataset.

| Dataset | Original | After masking |
| --- | --- | --- |
| *C.elegans* | 117 | 117 |
| *Mouse* | 224 | 223 |
| *New human* | 444 | 440 |
| *All_miRNA* | 1359 | 1255 |

**Table G.** Accuracy in classification of *All-miRNA* dataset after masking to remove homologs.

| Method | Original | | After masking | |
| --- | --- | --- | --- | --- |
|  | Sen | Spe | Sen | Spe |
| OC-SVM | 0.67 | 0.91 | 0.68 | 0.90 |
| OC-Gaussian | 0.82 | 0.99 | 0.87 | 0.97 |
| OC-Kmeans | 0.89 | 0.79 | 0.89 | 0.78 |
| OC-PCA | 0.90 | 0.85 | 0.90 | 0.85 |
| OC-KNN | 0.90 | 0.93 | 0.90 | 0.94 |

**Table H.** One-Class results obtained from the secondary features only and secondary features plus

sequence features. Sen = sensitivity and Spe = specificity. Results are presented for two genomes individually (*C.elegans and Mouse*) and *All-miRNA* as a mixture of multiple miRNAs species.

| Method | *C.elegans* | | | | *Mouse* | | | | *All-miRNA* | | | |
| --- | --- | --- | --- | --- | --- | --- | --- | --- | --- | --- | --- | --- |
|  | Secondary | | Secondary&  Sequence | | Secondary | | Secondary& Sequence | | Secondary | | Secondary& Sequence | |
|  | Sen | Spe | Sen | Spe | Sen | Spe | Sen | Spe | Sen | Spe | Sen | Spe |
| OC-SVM | 0.64 | 0.96 | 0.73 | 0.93 | 0.76 | 0.96 | 0.80 | 0.93 | 0.73 | 0.91 | 0.69 | 0.91 |
| OC-Gaussian | 0.75 | 0.97 | 0.84 | 0.93 | 0.84 | 0.97 | 0.89 | 0.93 | 0.88 | 0.90 | 0.82 | 0.99 |
| OC-Kmeans | 0.79 | 0.95 | 0.79 | 0.92 | 0.82 | 0.93 | 0.85 | 0.92 | 0.88 | 0.76 | 0.90 | 0.79 |
| OC-PCA | 0.88 | 0.87 | 0.87 | 0.89 | 0.89 | 0.92 | 0.88 | 0.92 | 0.90 | 0.83 | 0.90 | 0.85 |
| OC-KNN | 0.90 | 0.83 | 0.90 | 0.86 | 0.90 | 0.89 | 0.90 | 0.92 | 0.91 | 0.84 | 0.90 | 0.93 |
